# Supplementary material for: Opioid use as a potential risk factor for pancreatic cancer in the United States: An analysis of state and national level databases
Source: PLoS One. 2021 Jan 6;16(1):e0244285. doi: 10.1371/journal.pone.0244285 (PMC7787381; doi:10.1371/journal.pone.0244285)
Supplement: S1 Table — (DOCX) [file pone.0244285.s001.docx]

S1 Table: Pancreatic Cancer Incidence Rate by State, United States Cancer Statistics: Data Visualizations

|  | 1999 |  |  |  | 2016 |  |  |
| --- | --- | --- | --- | --- | --- | --- | --- |
| State | Incidence | LCI | UCI |  | Incidence | LCI | UCI |
| Alabama | 11.2 | 10.3 | 12.2 |  | 13.6 | 12.6 | 14.5 |
| Alaska | 13.6 | 9.5 | 18.6 |  | 14.7 | 11.7 | 18.2 |
| Arizona | 8.8 | 8.0 | 9.7 |  | 11.9 | 11.2 | 12.7 |
| Arkansas | - | - | - |  | 12.6 | 11.5 | 13.8 |
| California | 10.4 | 10.0 | 10.8 |  | 11.3 | 11.0 | 11.7 |
| Colorado | 9.8 | 8.8 | 10.9 |  | 11.6 | 10.7 | 12.5 |
| Connecticut | 11.8 | 10.7 | 13.0 |  | 14.3 | 13.2 | 15.5 |
| Delaware | 11.7 | 9.5 | 14.4 |  | 13.1 | 11.1 | 15.3 |
| District of Columbia | 14.7 | 11.6 | 18.2 |  | 13.0 | 10.4 | 16.1 |
| Florida | 11.1 | 10.7 | 11.6 |  | 12.5 | 12.1 | 12.9 |
| Georgia | 10.4 | 9.7 | 11.3 |  | 13.0 | 12.3 | 13.7 |
| Hawaii | 11.7 | 9.8 | 13.8 |  | 13.5 | 11.8 | 15.3 |
| Idaho | 11.5 | 9.6 | 13.6 |  | 12.9 | 11.3 | 14.6 |
| Illinois | 12.1 | 11.4 | 12.7 |  | 13.3 | 12.7 | 13.9 |
| Indiana | 11.0 | 10.2 | 11.9 |  | 13.5 | 12.7 | 14.4 |
| Iowa | 9.8 | 8.7 | 10.9 |  | 13.2 | 12.0 | 14.4 |
| Kansas | 9.6 | 8.5 | 10.9 |  | 12.0 | 10.8 | 13.2 |
| Kentucky | 10.7 | 9.7 | 11.7 |  | 12.9 | 11.9 | 13.9 |
| Louisiana | 12.5 | 11.4 | 13.6 |  | 13.6 | 12.6 | 14.6 |
| Maine | 12.3 | 10.5 | 14.3 |  | 14.0 | 12.3 | 15.9 |
| Maryland | 10.9 | 10.0 | 11.9 |  | 13.7 | 12.8 | 14.6 |
| Massachusetts | 11.8 | 11.0 | 12.7 |  | 11.1 | 10.4 | 11.8 |
| Michigan | 11.9 | 11.2 | 12.6 |  | 12.8 | 12.2 | 13.5 |
| Minnesota | 8.4 | 7.6 | 9.3 |  | 13.7 | 12.8 | 14.7 |
| Mississippi | - | - | - |  | 14.2 | 13.0 | 15.5 |
| Missouri | 11.8 | 10.9 | 12.7 |  | 11.9 | 11.1 | 12.7 |
| Montana | 10.3 | 8.3 | 12.5 |  | 12.9 | 11.0 | 15.1 |
| Nebraska | 10.2 | 8.8 | 11.8 |  | 14.2 | 12.6 | 15.9 |
| Nevada | 10.1 | 8.7 | 11.7 |  | 11.7 | 10.6 | 13.0 |
| New Hampshire | 11.6 | 9.7 | 13.7 |  | 12.9 | 11.2 | 14.8 |
| New Jersey | 12.5 | 11.8 | 13.3 |  | 13.4 | 12.7 | 14.1 |
| New Mexico | 11.1 | 9.5 | 12.8 |  | 11.2 | 9.9 | 12.6 |
| New York | 12.8 | 12.3 | 13.3 |  | 14.2 | 13.7 | 14.7 |
| North Carolina | 10.3 | 9.6 | 11.1 |  | 12.7 | 12.1 | 13.4 |
| North Dakota | 8.5 | 6.5 | 11.0 |  | 12.5 | 10.1 | 15.2 |
| Ohio | 10.6 | 10.0 | 11.2 |  | 12.0 | 11.4 | 12.6 |
| Oklahoma | 10.6 | 9.6 | 11.8 |  | 10.5 | 9.6 | 11.5 |
| Oregon | 11.7 | 10.6 | 12.9 |  | 11.7 | 10.8 | 12.8 |
| Pennsylvania | 11.2 | 10.7 | 11.8 |  | 14.3 | 13.7 | 14.9 |
| Rhode Island | 10.4 | 8.6 | 12.4 |  | 13.9 | 11.9 | 16.2 |
| South Carolina | 11.0 | 9.9 | 12.1 |  | 14.0 | 13.1 | 15.0 |
| 'South Dakota' | - | - | - |  | 14.1 | 11.9 | 16.7 |
| Tennessee | 7.7 | 7.0 | 8.5 |  | 12.6 | 11.8 | 13.4 |
| Texas | 10.3 | 9.9 | 10.8 |  | 12.5 | 12.1 | 12.9 |
| Utah | 9.2 | 7.7 | 10.8 |  | 9.8 | 8.6 | 11.1 |
| Vermont | 11.7 | 9.2 | 14.8 |  | 11.8 | 9.6 | 14.4 |
| Virginia | 8.5 | 7.8 | 9.2 |  | 12.5 | 11.8 | 13.2 |
| Washington | 11.6 | 10.7 | 12.5 |  | 13.0 | 12.2 | 13.8 |
| West Virginia | 8.6 | 7.4 | 10.0 |  | 12.1 | 10.7 | 13.6 |
| Wisconsin | 11.7 | 10.9 | 12.7 |  | 12.7 | 11.8 | 13.5 |
| Wyoming | 12.2 | 9.2 | 15.8 |  | 10.7 | 8.4 | 13.6 |

*(Per 100,000 people)

LCI: Lower Confidence Interval

UCI: Upper Confidence Interval

(Insufficient data characterized by “-”)
