# Supplementary material for: Opioid use as a potential risk factor for pancreatic cancer in the United States: An analysis of state and national level databases
Source: PLoS One. 2021 Jan 6;16(1):e0244285. doi: 10.1371/journal.pone.0244285 (PMC7787381; doi:10.1371/journal.pone.0244285)
Supplement: S2 Table — (DOCX) [file pone.0244285.s002.docx]

S2 Table: Opioid Death Rate by State, United States Cancer Statistics: Data Visualizations

|  | 1999 |  | 2016 |
| --- | --- | --- | --- |
| State | Mortality |  | Mortality |
| Alabama | 7.5 |  | 0.8 |
| Alaska | 12.5 |  | 4.0 |
| Arizona | 11.4 |  | 4.7 |
| Arkansas | 5.9 |  | 1.1 |
| California | 4.9 |  | 4.5 |
| Colorado | 9.5 |  | 3.7 |
| Connecticut | 24.5 |  | 4.4 |
| Delaware | 16.9 |  | 4.1 |
| District of Columbia | 30.0 |  | 4.9 |
| Florida | 14.4 |  | 2.6 |
| Georgia | 8.8 |  | 1.0 |
| Hawaii | 5.2 |  | 2.4 |
| Idaho | 7.4 |  | 2.5 |
| Illinois | 15.3 |  | 3.9 |
| Indiana | 12.6 |  | 0.8 |
| Iowa | 6.2 |  | - |
| Kansas | 5.1 |  | 1.0 |
| Kentucky | 23.6 |  | 1.3 |
| Louisiana | 7.7 |  | 0.7 |
| Maine | 25.2 |  | 2.1 |
| Maryland | 29.7 |  | 8.8 |
| Massachusetts | 29.7 |  | 5.0 |
| Michigan | 18.5 |  | 1.2 |
| Minnesota | 7.4 |  | 1.2 |
| Mississippi | 6.2 |  | - |
| Missouri | 15.9 |  | 1.8 |
| Montana | 4.2 |  | - |
| Nebraska | 2.4 |  | - |
| Nevada | 13.3 |  | 7.8 |
| New Hampshire | 35.8 |  | 3.2 |
| New Jersey | 16.0 |  | 3.8 |
| New Mexico | 17.5 |  | 10.2 |
| New York | 15.1 |  | 3.2 |
| North Carolina | 15.4 |  | 2.0 |
| North Dakota | 7.6 |  | - |
| Ohio | 32.9 |  | 1.5 |
| Oklahoma | 11.6 |  | 2.7 |
| Oregon | 7.6 |  | 3.5 |
| Pennsylvania | 18.5 |  | 2.7 |
| Rhode Island | 26.7 |  | 3.4 |
| South Carolina | 13.1 |  | 1.2 |
| 'South Dakota' | 5.0 |  | - |
| Tennessee | 18.1 |  | 1.6 |
| Texas | 4.9 |  | 1.7 |
| Utah | 16.4 |  | 7.3 |
| Vermont | 18.4 |  | - |
| Virginia | 13.5 |  | 2.7 |
| Washington | 9.4 |  | 5.9 |
| West Virginia | 43.4 |  | 1.8 |
| Wisconsin | 15.8 |  | 1.6 |
| Wyoming | 8.7 |  | - |

*(Per 100,000 people)

(Insufficient data characterized by “-”)
