# Supplementary material for: Opioid use as a potential risk factor for pancreatic cancer in the United States: An analysis of state and national level databases
Source: PLoS One. 2021 Jan 6;16(1):e0244285. doi: 10.1371/journal.pone.0244285 (PMC7787381; doi:10.1371/journal.pone.0244285)
Supplement: S3 Table — (DOCX) [file pone.0244285.s003.docx]

S3 Table: Obesity Prevalence by State (%), Behavioral Risk Factor Surveillance System (BRFSS)

|  | 1999 |  |  |  | 2018 |  |  |
| --- | --- | --- | --- | --- | --- | --- | --- |
| State | Prevalence (%) | LCI (%) | UCI (%) |  | Prevalence (%) | LCI (%) | UCI (%) |
| Alabama | 22.4 | 20.3 | 24.5 |  | 36.2 | 34.6 | 37.9 |
| Alaska | 20.4 | 18.0 | 22.8 |  | 29.5 | 26.9 | 32.1 |
| Arizona | 12.3 | 10.1 | 14.5 |  | 29.5 | 27.8 | 31.2 |
| Arkansas | 22.7 | 21.0 | 24.4 |  | 37.1 | 35.1 | 39.1 |
| California | 18.7 | 17.3 | 20.1 |  | 25.8 | 24.8 | 26.9 |
| Colorado | 14.9 | 13.1 | 16.7 |  | 23 | 21.9 | 24 |
| Connecticut | 15.1 | 13.5 | 16.7 |  | 27.4 | 26.2 | 28.6 |
| Delaware | 17.5 | 15.5 | 19.5 |  | 33.5 | 31.7 | 35.3 |
| District of Columbia | 18.5 | 16.1 | 20.9 |  | 24.7 | 22.9 | 26.5 |
| Florida | 18.6 | 17.3 | 19.9 |  | 30.7 | 29.1 | 32.2 |
| Georgia | 21.1 | 19.1 | 23.1 |  | 32.5 | 31.2 | 33.7 |
| Hawaii | 15.7 | 13.6 | 17.8 |  | 24.9 | 23.6 | 26.3 |
| Idaho | 20.0 | 18.6 | 21.4 |  | 28.4 | 26.3 | 30.4 |
| Illinois | 20.9 | 19.1 | 22.7 |  | 31.8 | 30.2 | 33.3 |
| Indiana | 19.9 | 17.3 | 22.5 |  | 34.1 | 32.6 | 35.5 |
| Iowa | 21.5 | 19.9 | 23.1 |  | 35.3 | 34.1 | 36.5 |
| Kansas | 18.9 | 17.5 | 20.3 |  | 34.4 | 33.2 | 35.6 |
| Kentucky | 21.7 | 20.3 | 23.1 |  | 36.6 | 34.9 | 38.4 |
| Louisiana | 22.3 | 20.0 | 24.6 |  | 36.8 | 34.9 | 38.6 |
| Maine | 19.4 | 17.2 | 21.6 |  | 30.4 | 28.9 | 31.8 |
| Maryland | 18.2 | 16.7 | 19.7 |  | 30.9 | 29.8 | 32.1 |
| Massachusetts | 14.7 | 13.4 | 16.0 |  | 25.7 | 24.2 | 27.1 |
| Michigan | 22.8 | 20.9 | 24.7 |  | 33.0 | 31.8 | 34.2 |
| Minnesota | 15.5 | 14.4 | 16.6 |  | 30.1 | 29.2 | 30.9 |
| Mississippi | 23.2 | 21.0 | 25.4 |  | 39.5 | 37.8 | 41.2 |
| Missouri | 21.7 | 20.0 | 23.4 |  | 35.0 | 33.3 | 36.8 |
| Montana | 15.8 | 13.9 | 17.7 |  | 26.9 | 25.2 | 28.5 |
| Nebraska | 21.0 | 19.3 | 22.7 |  | 34.1 | 32.9 | 35.3 |
| Nevada | 15.8 | 13.5 | 18.1 |  | 29.5 | 27.1 | 31.9 |
| New Hampshire | 14.6 | 12.3 | 16.9 |  | 29.6 | 27.8 | 31.4 |
| New Jersey | 17.0 | 15.2 | 18.8 |  | 25.7 | 23.2 | 28.1 |
| New Mexico | 17.7 | 16.3 | 19.1 |  | 32.3 | 30.7 | 34.0 |
| New York | 17.4 | 15.7 | 19.1 |  | 27.6 | 26.7 | 28.5 |
| North Carolina | 21.5 | 19.6 | 23.4 |  | 33.0 | 31.2 | 34.8 |
| North Dakota | 21.9 | 19.9 | 23.9 |  | 35.1 | 33.2 | 37.0 |
| Ohio | 20.3 | 18.0 | 22.6 |  | 34.0 | 32.7 | 35.3 |
| Oklahoma | 21.1 | 19.4 | 22.8 |  | 34.8 | 33.1 | 36.6 |
| Oregon | 19.9 | 17.9 | 21.9 |  | 29.9 | 28.4 | 31.4 |
| Pennsylvania | 20.3 | 18.8 | 21.8 |  | 30.9 | 29.4 | 32.5 |
| Rhode Island | 16.8 | 15.4 | 18.2 |  | 27.7 | 26.0 | 29.5 |
| South Carolina | 20.6 | 19.0 | 22.2 |  | 34.3 | 33.0 | 35.6 |
| 'South Dakota' | 19.6 | 18.2 | 21.0 |  | 30.1 | 28.0 | 32.1 |
| Tennessee | 20.5 | 18.8 | 22.2 |  | 34.4 | 32.5 | 36.3 |
| Texas | 21.6 | 20.1 | 23.1 |  | 34.8 | 32.8 | 36.8 |
| Utah | 16.7 | 14.9 | 18.5 |  | 27.8 | 26.7 | 28.9 |
| Vermont | 18.0 | 16.4 | 19.6 |  | 27.5 | 25.9 | 29.2 |
| Virginia | 19.3 | 17.4 | 21.2 |  | 30.4 | 29.1 | 31.6 |
| Washington | 18.2 | 16.7 | 19.7 |  | 28.7 | 27.6 | 29.7 |
| West Virginia | 24.6 | 22.7 | 26.5 |  | 39.5 | 37.8 | 41.3 |
| Wisconsin | 19.9 | 18.0 | 21.8 |  | 32.0 | 30.2 | 33.8 |
| Wyoming | 16.9 | 15.3 | 18.5 |  | 29.0 | 27.3 | 30.8 |

*(BMI) ≥ 30.0

LCI: Lower Confidence Interval

UCI: Upper Confidence Interval

(Insufficient data characterized by “-”)
