# Supplementary material for: Opioid use as a potential risk factor for pancreatic cancer in the United States: An analysis of state and national level databases
Source: PLoS One. 2021 Jan 6;16(1):e0244285. doi: 10.1371/journal.pone.0244285 (PMC7787381; doi:10.1371/journal.pone.0244285)
Supplement: S5 Table — (DOCX) [file pone.0244285.s005.docx]

S5 Table: Cigarette Use by State (%), Behavioral Risk Factor Surveillance System (BRFSS)

|  | 1999 |  |  |  | 2017 |  |  |
| --- | --- | --- | --- | --- | --- | --- | --- |
| State | Prevalence (%) | LCI (%) | UCI (%) |  | Prevalence (%) | LCI (%) | UCI (%) |
| Alabama | 23.5 | 21.4 | 25.6 |  | 20.9 | 19.5 | 22.4 |
| Alaska | 27.3 | 24.4 | 30.2 |  | 21 | 18.3 | 23.7 |
| Arizona | 20.1 | 17.3 | 22.9 |  | 15.6 | 14.8 | 16.4 |
| Arkansas | 27.2 | 25.4 | 29.0 |  | 22.3 | 20.0 | 24.5 |
| California | 18.7 | 17.4 | 20.0 |  | 11.3 | 10.4 | 12.3 |
| Colorado | 22.5 | 20.4 | 24.6 |  | 14.6 | 13.7 | 15.6 |
| Connecticut | 22.8 | 20.6 | 25.0 |  | 12.7 | 11.7 | 13.7 |
| Delaware | 25.4 | 22.9 | 27.9 |  | 17.0 | 15.3 | 18.7 |
| District of Columbia | 20.6 | 18 | 23.2 |  | 14.4 | 12.9 | 15.8 |
| Florida | 20.6 | 19.3 | 21.9 |  | 16.1 | 14.9 | 17.3 |
| Georgia | 23.7 | 21.6 | 25.8 |  | 17.5 | 16.1 | 18.8 |
| Hawaii | 18.5 | 16.3 | 20.7 |  | 12.8 | 11.7 | 13.9 |
| Idaho | 21.5 | 20.1 | 22.9 |  | 14.4 | 12.9 | 15.8 |
| Illinois | 24.2 | 22.4 | 26.0 |  | 15.5 | 14.2 | 16.8 |
| Indiana | 27.0 | 24.0 | 30.0 |  | 21.8 | 20.8 | 22.8 |
| Iowa | 23.5 | 21.8 | 25.2 |  | 17.1 | 16.0 | 18.1 |
| Kansas | 21.0 | 19.5 | 22.5 |  | 17.4 | 16.7 | 18.1 |
| Kentucky | 29.7 | 28.2 | 31.2 |  | 24.6 | 23.0 | 26.2 |
| Louisiana | 23.5 | 21.1 | 25.9 |  | 23.1 | 21.4 | 24.7 |
| Maine | 23.3 | 20.8 | 25.8 |  | 17.3 | 16.0 | 18.6 |
| Maryland | 20.3 | 18.7 | 21.9 |  | 13.9 | 12.9 | 14.9 |
| Massachusetts | 19.3 | 17.9 | 20.7 |  | 13.7 | 12.3 | 15.0 |
| Michigan | 25.1 | 23.2 | 27.0 |  | 19.3 | 18.3 | 20.3 |
| Minnesota | 19.5 | 18.3 | 20.7 |  | 14.5 | 13.8 | 15.3 |
| Mississippi | 22.9 | 20.9 | 24.9 |  | 22.2 | 20.4 | 24.0 |
| Missouri | 27.1 | 25.2 | 29.0 |  | 20.8 | 19.4 | 22.1 |
| Montana | 20.2 | 18.1 | 22.3 |  | 17.2 | 15.8 | 18.6 |
| Nebraska | 23.2 | 21.4 | 25.0 |  | 15.4 | 14.5 | 16.3 |
| Nevada | 31.5 | 28.5 | 34.5 |  | 17.6 | 15.6 | 19.6 |
| New Hampshire | 22.3 | 19.6 | 25.0 |  | 15.7 | 14.1 | 17.3 |
| New Jersey | 20.6 | 18.7 | 22.5 |  | 13.7 | 12.6 | 14.9 |
| New Mexico | 22.4 | 20.9 | 23.9 |  | 17.5 | 16.1 | 18.9 |
| New York | 21.8 | 20.0 | 23.6 |  | 14.1 | 13.2 | 15.0 |
| North Carolina | 25.1 | 23.0 | 27.2 |  | 17.2 | 15.7 | 18.7 |
| North Dakota | 22.1 | 20.1 | 24.1 |  | 18.3 | 17.0 | 19.6 |
| Ohio | 27.6 | 25.0 | 30.2 |  | 21.1 | 20.0 | 22.3 |
| Oklahoma | 25.2 | 23.3 | 27.1 |  | 20.2 | 18.8 | 21.5 |
| Oregon | 21.4 | 19.3 | 23.5 |  | 16.1 | 14.8 | 17.3 |
| Pennsylvania | 23.1 | 21.5 | 24.7 |  | 18.8 | 17.5 | 20.0 |
| Rhode Island | 22.3 | 20.8 | 23.8 |  | 15.0 | 13.4 | 16.5 |
| South Carolina | 23.6 | 21.9 | 25.3 |  | 18.8 | 17.7 | 19.9 |
| 'South Dakota' | 22.5 | 21.0 | 24.0 |  | 19.3 | 17.3 | 21.2 |
| Tennessee | 24.8 | 23.0 | 26.6 |  | 22.6 | 21.0 | 24.3 |
| Texas | 22.4 | 20.8 | 24.0 |  | 15.7 | 14.3 | 17.2 |
| Utah | 14.0 | 12.4 | 15.6 |  | 8.9 | 8.1 | 9.6 |
| Vermont | 21.7 | 20.0 | 23.4 |  | 15.8 | 14.5 | 17.1 |
| Virginia | 21.4 | 19.6 | 23.2 |  | 16.4 | 15.3 | 17.5 |
| Washington | 22.4 | 20.7 | 24.1 |  | 13.5 | 12.7 | 14.3 |
| West Virginia | 27.1 | 25.1 | 29.1 |  | 26.0 | 24.5 | 27.5 |
| Wisconsin | 23.7 | 21.7 | 25.7 |  | 16.0 | 14.7 | 17.4 |
| Wyoming | 23.9 | 21.9 | 25.9 |  | 18.7 | 17.2 | 20.3 |

* Current smoker status

LCI: Lower Confidence Interval

UCI: Upper Confidence Interval

(Insufficient data characterized by “-”)
