# Supplementary material for: Opioid use as a potential risk factor for pancreatic cancer in the United States: An analysis of state and national level databases
Source: PLoS One. 2021 Jan 6;16(1):e0244285. doi: 10.1371/journal.pone.0244285 (PMC7787381; doi:10.1371/journal.pone.0244285)
Supplement: S6 Table — (DOCX) [file pone.0244285.s006.docx]

S6 Table: Mixed Effects Regression Model Using Log Transformed Opioid Death Rate

| **Four-year lagged risk factors** | **Estimate** | **Standard Error** | **P-Value** |
| --- | --- | --- | --- |
| Opioid death rate (Log) | 1.068 | 0.248 | < .0001 |
| Obesity prevalence | 0.027 | 0.035 | 0.438 |
| Alcohol prevalence | 0.025 | 0.009 | 0.006 |
| Cigarette use prevalence | -0.016 | 0.018 | 0.383 |
| Time | 0.092 | 0.021 | < .0001 |
|  |  |  |  |
| **Interactions with risk factors with time^*^** | **Estimate** | **Standard Error** | **P-Value** |
| Opioid death rate* (Log) | -0.115 | 0.023 | < .0001 |
| Obesity prevalence* | 0.002 | 0.002 | 0.352 |
| Interaction between opioid death rate (Log) and obesity prevalence | 0.083 | 0.030 | 0.005 |

* Denotes that this variable interacted with time in the statistical model.
