# Supplementary figures and images for: Opioid use as a potential risk factor for pancreatic cancer in the United States: An analysis of state and national level databases
Source: PLoS One. 2021 Jan 6;16(1):e0244285. doi: 10.1371/journal.pone.0244285 (PMC7787381; doi:10.1371/journal.pone.0244285)

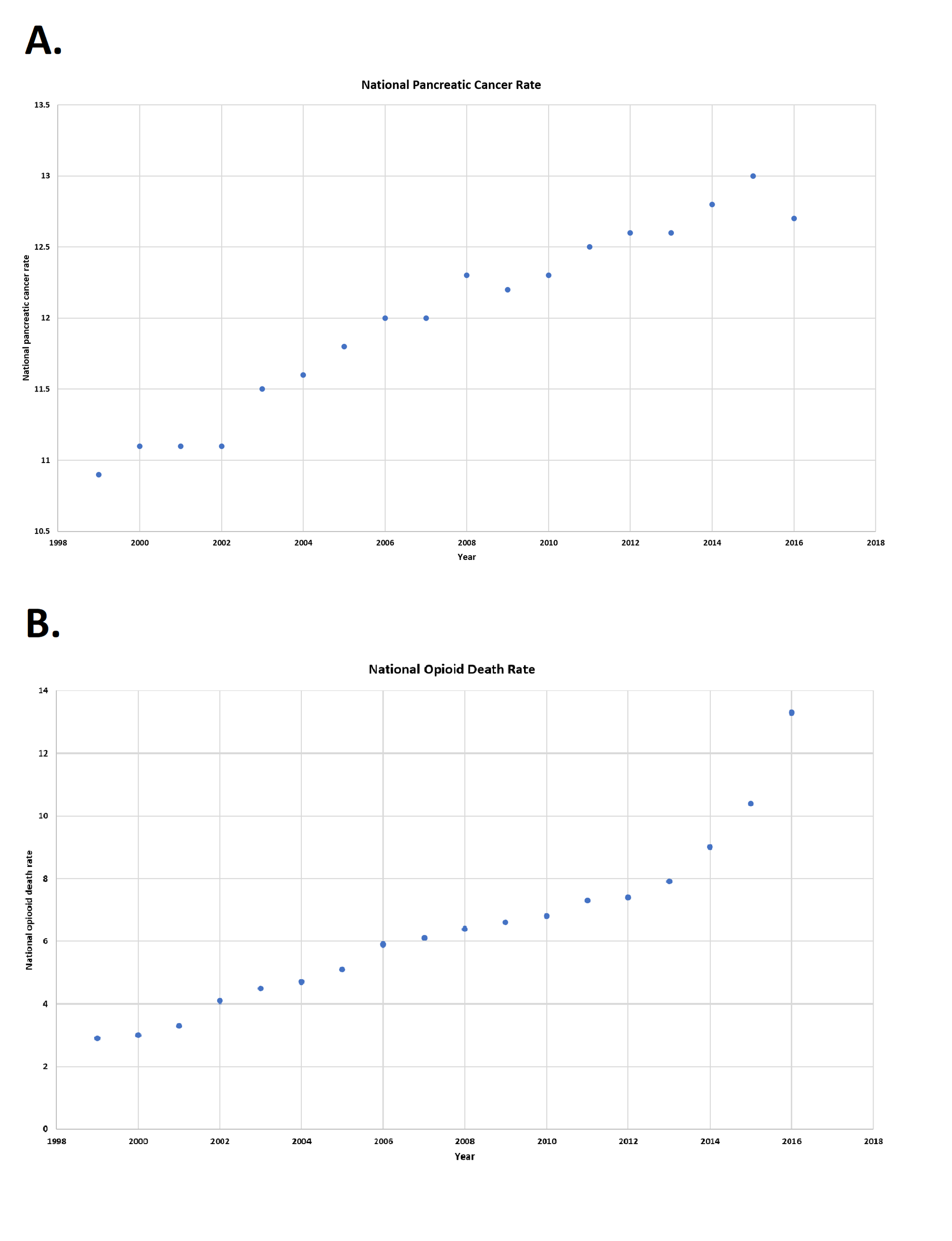

Supplement: S1 Fig — (A) Incidence rate of pancreatic cancer over time per 100,000 people, National incidence rate of pancreatic cancer through the years 1999 to 2016, (B) Incidence rate of opioid death overtime per 100,000 people, National incidence rate of opioid death rate through the years 1999 to 2016. (TIF) [file pone.0244285.s007.tif]

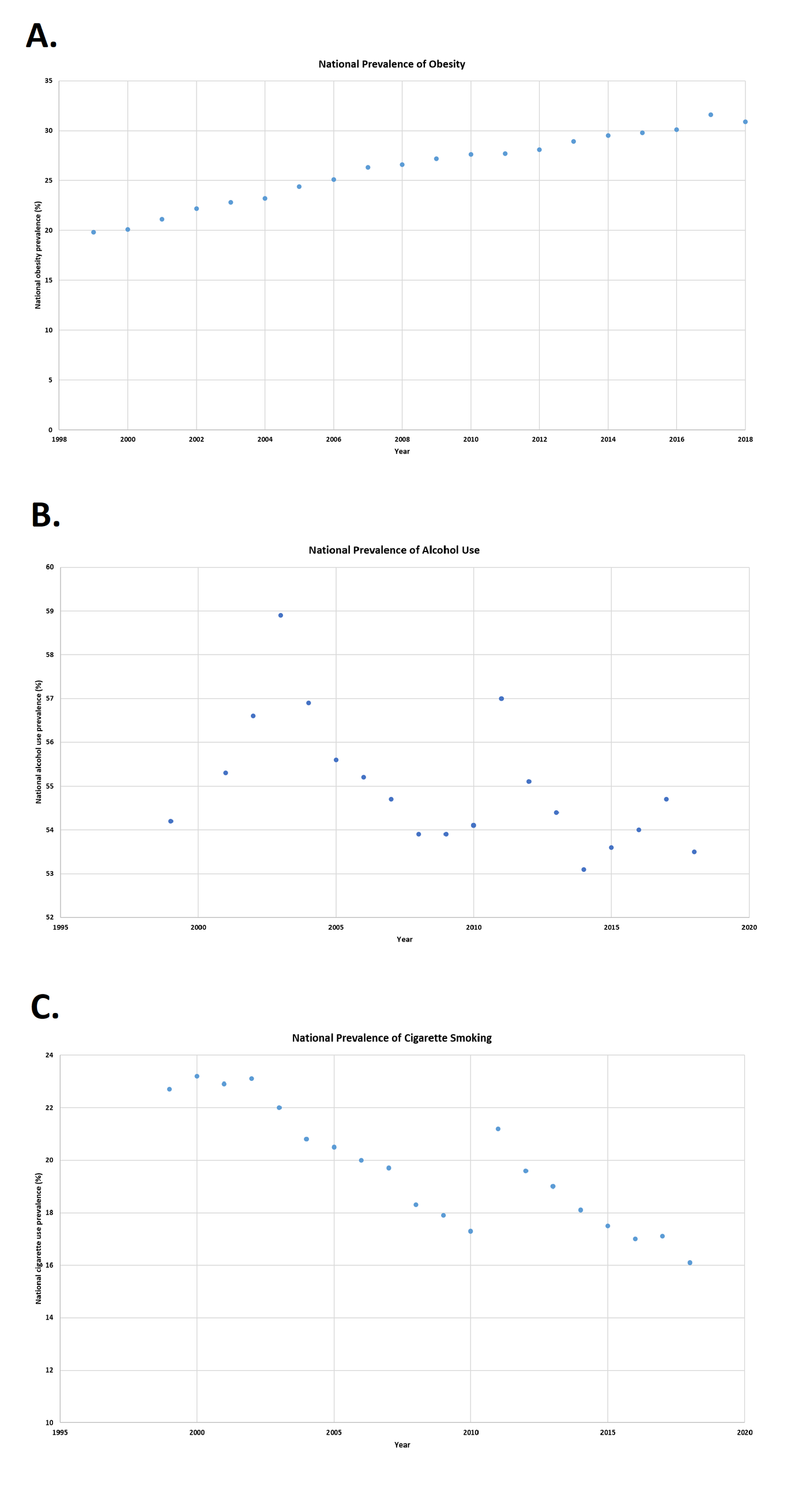

Supplement: S2 Fig — (A) National prevalence of obesity through the years 1999 to 2018, (B) National prevalence (%) of alcohol use through the years 1999 to 2018, (C) National prevalence (%) of tobacco use through the years 1999 to 2017. (TIF) [file pone.0244285.s008.tif]

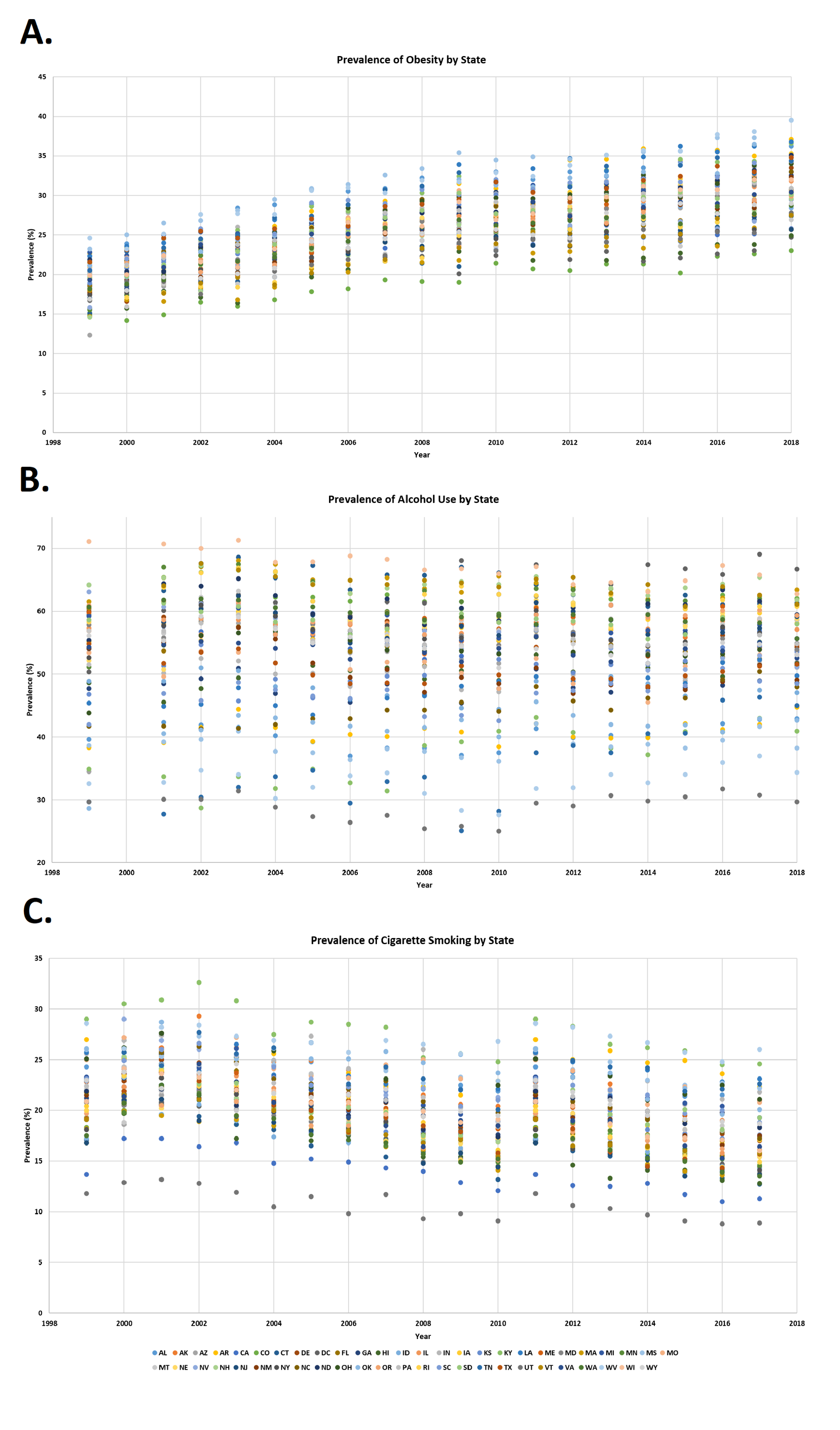

Supplement: S3 Fig — (A) State prevalence of obesity overtime, Prevalence of obesity by state through the years 1999 to 2018, (B) Prevalence of alcohol use by state through the years 1999 to 2018, (C) Prevalence of tobacco use by state through the years 1999 to 2017. (TIF) [file pone.0244285.s009.tif]
