# Supplementary material for: Opioid use as a potential risk factor for pancreatic cancer in the United States: An analysis of state and national level databases
Source: PLoS One. 2021 Jan 6;16(1):e0244285. doi: 10.1371/journal.pone.0244285 (PMC7787381; doi:10.1371/journal.pone.0244285)
Supplement: Attachment — Submitted filename: PONE-D-20-20071_reviewer (1).pdf [file pone.0244285.s010.pdf]

PLOS ONE

Opioid use as a potential contributing factor to the rise of pancreatic cancer; an analysis of state and national level databases from the United States

--Manuscript Draft--

**Manuscript Number:**	PONE-D-20-20071
**Article Type:**	Research Article
**Full Title:**	Opioid use as a potential contributing factor to the rise of pancreatic cancer; an analysis of state and national level databases from the United States
**Short Title:**	Opioid use and pancreas cancer
**Corresponding Author:**	Faraz Bishehsari<br><br>UNITED STATES
**Keywords:**	Pancreas; cancer; opioids; Alcohol
**Abstract:**	Pancreatic cancer (PC) rate is increasing in the U.S. The use of prescription and illicit opioids has continued to rise nationally in recent years as well. Opioids have been shown to have a deleterious effect on multiple types of cancer with recent data suggesting opium use as a risk factor for PC. Using national databases, we tested whether opioid usage pattern over time could explain the state and national-based variations in PC rates in the U.S.<br>Opioid death rate (as a surrogate for prescription and illicit opioid use) was extracted from the CDCs Wonder online data through the Vital Statistics Cooperative Program. Incidence of pancreatic cancer was retrieved from the online CDCs data base gathered from the U.S. Cancer Statistics Working Group. Prevalence of obesity, tobacco and alcohol use was collected from Behavioral risk factor surveillance system. Mixed-effects regression models were used to test the association between levels of PC rate and opioid death/use rates during the years 1999-2016.<br>A rise in PC was seen over time at the national and state levels. Similarly, the opioid death rates increased over time. Among other potential PC risk factors, only obesity prevalence showed an increase during the study period. The initial level of a state's opioid death rate significantly predicted incidence of PC (β=0.1848, p<0.0001) and had a significant effect on the estimated annual change in the rate of PC (β=-.0193, p<0.0001).<br>Opioid use may be an un-identified risk factor contributing to the increasing incidence of PC in the U.S. These novel findings need to be verified by population-based studies.
**Order of Authors:**	Usman Barlass
	Ameya Ashutosh Deshmukh, B.A.
	Todd Beck
	Faraz Bishehsari
**Additional Information:**	
**Question**	**Response**
**Financial Disclosure**<br><br>Enter a financial disclosure statement that describes the sources of funding for the work included in this submission. Review the submission guidelines for detailed requirements. View published research articles from *PLOS ONE* for specific examples.<br><br>This statement is required for submission	F.B. is supported by grants from the Rush Translational Sciences Consortium/Swim Across America Organization, Brinson Foundation and Institute for Translational Medicine (ITM), the National Center for Advancing Translational Sciences of the National Institutes of Health (NIH) (grant No. 5UL1TR002389–02) that funds the ITM

Powered by Editorial Manager® and ProduXion Manager® from Aries Systems Corporation

and **will appear in the published article** if the submission is accepted. Please make sure it is accurate.<br><br>**Unfunded studies**<br>Enter: *The author(s) received no specific funding for this work.*<br><br>**Funded studies**<br>Enter a statement with the following details:<br>• Initials of the authors who received each award<br>• Grant numbers awarded to each author<br>• The full name of each funder<br>• URL of each funder website<br>• Did the sponsors or funders play any role in the study design, data collection and analysis, decision to publish, or preparation of the manuscript?<br>• **NO** - Include this sentence at the end of your statement: *The funders had no role in study design, data collection and analysis, decision to publish, or preparation of the manuscript.*<br>• **YES** - Specify the role(s) played.<br><br>* typeset	
**Competing Interests**<br><br>Use the instructions below to enter a competing interest statement for this submission. On behalf of all authors, disclose any competing interests that could be perceived to bias this work—acknowledging all financial support and any other relevant financial or non-financial competing interests.<br><br>This statement **will appear in the published article** if the submission is accepted. Please make sure it is accurate. View published research articles from *PLOS ONE* for specific examples.	The authors have declared that no competing interests exist.

**NO authors have competing interests**<br><br>Enter: *The authors have declared that no competing interests exist.*<br><br>**Authors with competing interests**<br><br>Enter competing interest details beginning with this statement:<br><br>*I have read the journal's policy and the authors of this manuscript have the following competing interests: [insert competing interests here]*<br><br>* typeset	
**Ethics Statement**<br><br>Enter an ethics statement for this submission. This statement is required if the study involved:<br><br>• Human participants<br>• Human specimens or tissue<br>• Vertebrate animals or cephalopods<br>• Vertebrate embryos or tissues<br>• Field research<br><br>Write "N/A" if the submission does not require an ethics statement.<br><br>General guidance is provided below. Consult the submission guidelines for detailed instructions. **Make sure that all information entered here is included in the Methods section of the manuscript.**	N/A

Powered by Editorial Manager® and ProduXion Manager® from Aries Systems Corporation

**Format for specific study types**<br><br>**Human Subject Research (involving human participants and/or tissue)**<br>• Give the name of the institutional review board or ethics committee that approved the study<br>• Include the approval number and/or a statement indicating approval of this research<br>• Indicate the form of consent obtained (written/oral) or the reason that consent was not obtained (e.g. the data were analyzed anonymously)<br><br>**Animal Research (involving vertebrate animals, embryos or tissues)**<br>• Provide the name of the Institutional Animal Care and Use Committee (IACUC) or other relevant ethics board that reviewed the study protocol, and indicate whether they approved this research or granted a formal waiver of ethical approval<br>• Include an approval number if one was obtained<br>• If the study involved *non-human primates*, add *additional details* about animal welfare and steps taken to ameliorate suffering<br>• If anesthesia, euthanasia, or any kind of animal sacrifice is part of the study, include briefly which substances and/or methods were applied<br><br>**Field Research**<br><br>Include the following details if this study involves the collection of plant, animal, or other materials from a natural setting:<br>• Field permit number<br>• Name of the institution or relevant body that granted permission	
**Data Availability**<br><br>Authors are required to make all data underlying the findings described fully available, without restriction, and from the time of publication. PLOS allows rare exceptions to address legal and ethical concerns. See the PLOS Data Policy and FAQ for detailed information.	Yes - all data are fully available without restriction

A Data Availability Statement describing where the data can be found is required at submission. Your answers to this question constitute the Data Availability Statement and **will be published in the article**, if accepted.<br><br>**Important:** Stating 'data available on request from the author' is not sufficient. If your data are only available upon request, select 'No' for the first question and explain your exceptional situation in the text box.<br><br>Do the authors confirm that all data underlying the findings described in their manuscript are fully available without restriction?	
**Describe where the data may be found in full sentences. If you are copying our sample text, replace any instances of XXX with the appropriate details.**<br><br>• If the data are **held or will be held in a public repository**, include URLs, accession numbers or DOIs. If this information will only be available after acceptance, indicate this by ticking the box below. For example: *All XXX files are available from the XXX database (accession number(s) XXX, XXX.).*<br>• If the data are all contained **within the manuscript and/or Supporting Information files**, enter the following: *All relevant data are within the manuscript and its Supporting Information files.*<br>• If neither of these applies but you are able to provide **details of access elsewhere**, with or without limitations, please do so. For example:<br><br>*Data cannot be shared publicly because of [XXX]. Data are available from the XXX Institutional Data Access / Ethics Committee (contact via XXX) for researchers who meet the criteria for access to confidential data.*<br><br>*The data underlying the results presented in the study are available from (include the name of the third party*	All relevant data are within the manuscript and its Supporting Information files.

and contact information or URL).<br>• This text is appropriate if the data are owned by a third party and authors do not have permission to share the data.<br><br>* typeset	
Additional data availability information:	

Manuscript

Click here to access/download;Manuscript;Opioid use and pancreatic cancer-Revised - CL.docx

Opioid use as a potential contributing factor to the rise of pancreatic cancer; an analysis of state and national level databases from the United States

Barlass, Usman[1]*, Deshmukh Ameya[2]*, Beck Todd[3], Bishehsari, Faraz[1€]

1. Department of Internal Medicine, Division of Gastroenterology, Rush University Medical Center, Chicago, IL USA
2. Midwestern University – CCOM, Downers Grove, IL USA
3. Bioinformatics and Biostatistics Core, Rush University Medical Center, Chicago, IL USA

*These authors had equal contributions

Short title: Opioid use and pancreas cancer

Abbreviations: PC: Pancreatic Cancer; U.S : United States, CDC : Centers for Disease Control and Prevention

[€] **Correspondence to**:

Faraz Bishehsari, MD,PhD.

Associate Professor of Medicine and Graduate College

Division of Digestive Diseases, Rush University Medical Center Professional Building, 1725 W. Harrison St. Suite 207, Chicago, IL 60612, USA, Tel.: 11-312-563-4092, Fax: 11-312-563-3945

E-mail: faraz_bishehsari@rush.edu

Writing assistance: None

Grant support:

F.B. is supported by grants from the Rush Translational Sciences Consortium/Swim

Across America Organization, Brinson Foundation and Institute for Translational Medicine (ITM), the National Center for Advancing Translational Sciences of the National Institutes of Health (NIH) (grant No. 5UL1TR002389–02) that funds the ITM. The content is solely the responsibility of the authors and does not necessarily represent the official views of the NIH.

Author contributions:

Study concept and design: U.B, F.B

Analysis and interpretation of data: U.B, A.D, T.B, F.B

Drafting of manuscript: U.B, A.D, T.B, F.B

Critical revision of manuscript: U.B, A.D, T.B, F.B

Statistical analysis: AD, T.B.

Study supervision: F.B

Abstract

Pancreatic cancer (PC) rate is increasing in the U.S. The use of prescription and illicit opioids has continued to rise nationally in recent years as well. Opioids have been shown to have a deleterious effect on multiple types of cancer with recent data suggesting opium use as a risk factor for PC. Using national databases, we tested whether opioid usage pattern over time could explain the state and national-based variations in PC rates in the U.S.

Opioid death rate (as a surrogate for prescription and illicit opioid use) was extracted from the CDCs Wonder online data through the Vital Statistics Cooperative Program. Incidence of pancreatic cancer was retrieved from the online CDCs data base gathered from the U.S. Cancer Statistics Working Group. Prevalence of obesity, tobacco and alcohol use was collected from Behavioral risk factor surveillance system. Mixed-effects regression models were used to test the association between levels of PC rate and opioid death/use rates during the years 1999-2016.

A rise in PC was seen over time at the national and state levels. Similarly, the opioid death rates increased over time. Among other potential PC risk factors, only obesity prevalence showed an increase during the study period. The initial level of a state's opioid death rate significantly predicted incidence of PC ($\beta=0.1848$, $p<0.0001$) and had a significant effect on the estimated annual change in the rate of PC ($\beta=-.0193$, $p<0.0001$).

Opioid use may be an un-identified risk factor contributing to the increasing incidence of PC in the U.S. These novel findings need to be verified by population-based studies.

Key words: Pancreas, cancer, opioids, alcohol

Introduction

Pancreatic cancer (PC) is currently the third leading cause of cancer related mortality in the United States (U.S) (1). The five year survival rate for PC remains dismal (~10%), partly due to asymptomatic progression of the disease with a majority (~85%) of the patients having non-surgically resectable disease at the time of diagnosis (2). More alarming is the ongoing increase in the PC incidence which will place the disease as the second cause of cancer mortality by the end of this decade (3). Discovering risk factors contributing to PC can aid with identifying high risk groups that would benefit from implementation of preventive and possibly screening strategies, which could help in reduction of the disease burden and/or early diagnosis.

While the established risk factors for PC including genetic predisposition syndromes and chronic pancreatitis (alcohol or non-alcohol causes) only account for a small portion of PC incident cases, the etiology for the majority of cases has remained unknown. Several factors associated with our lifestyle habits (e.g., smoking) have been proposed to modulate the risk of PC (1, 4-6). Besides increasing obesity rates, exposure to a number of PC risk factors, such as smoking and alcohol, has been steady or even decreasing at the population level, contrary to the increase in the PC rates in the recent years. Therefore, novel factors may be at play.

Recent population based studies have suggested opium use to increase risk of PC in a dose dependent manner (7, 8). While opium use is not a common recreational habit in the U.S., opioid use has been rising remarkably over the past decade. In fact, opioid misuse and overdose have evolved into a public health crisis. Approximately 70,000 drug overdose deaths were reported in 2017, 68% of which involved an opioid (9). The use of prescription opioids for the management of chronic pain has increased remarkably with more than 191 million opioid prescriptions given to patients in the U.S in 2017. Not surprisingly, the opioid addiction rates among patients who are given opioid for chronic pain has increased with 29% of such patients misusing opioids, and 12% developing an opioid use disorder (10).

Using national datasets, we aimed to evaluate whether the trend in the burden of opioid usage could explain the trend in PC at the national and state levels overtime.

Methods

<u>Data collection</u>:

Pancreatic cancer incidence rates (age adjusted to the 2000 US standard population) from 1999 to 2016 for all 50 states and the District of Columbia and nationally were obtained from the CDC's U.S Cancer Statistics database (11) [illegible]is data was retrieved from the CDC's publicly available national database through their behavioral risk factor surveillance system (BRFSS) (supplementary tables 1 to [illegible]Upper and lower 95% confidence intervals (CIs) were retrieved for each risk factor from the existing datasets.

Opioid death rate data originated from the CDC wonder online database (released 2018) and retrieved through the Kaiser Family Foundation analysis (12). Opioid death rate was used as a surrogate marker for overall opioid use to account for both prescription and illicit opioid use. Data are from the Multiple Cause of Death Files, 1999-2018, through the Vital Statistics Cooperative Program, as compiled from data provided by the 57 vital statistics jurisdictions. Drug overdose deaths were classified using the International Classification of Disease, Tenth Revision (ICD-10).

In order to correct for other possible proposed risk factors for PC, current cigarette smoking prevalence (1999 to 2017), obesity prevalence (1999 to 2018) and alcohol use prevalence (1999 to 2018) were also collected (13). Obesity was defined as body mass index (BMI) ≥ 30.0, calculated from self-reported weight and height (weight [kg]/ height [m²]). Pregnant women and respondents reporting weight < 50 pounds or ≥ 650 pounds; height < 3 feet or ≥ 8 feet; or BMI: <12 or ≥ 100 were excluded. Alcohol use prevalence was determined from adults who ingested at least one alcoholic beverage in the past 30 days. All risk factors were based on using the full population (Tables 1-5)

Each variable data point at a specific year was linked to the PC rate four years later to account for the least potential lag time for the disease development from the time of exposure (14).

To illustrate trends, incidence rates and prevalence were plotted for each individual state from 1999 to 2016.

Statistical analysis:

Linear mixed-effects models allowing for state specific slopes and intercepts were used to assess the unadjusted time trends over the length of the study period in PC, opioid death rate, obesity, alcohol use and cigarette use. The average state trend was then plotted on a scatter plot of the raw statewide data for each variable of interest. A mixed-effects regression model was then used to examine the multivariate longitudinal association between the change of pancreatic cancer incidence rates and the risk factors included in this study for the years 1999-2016 (15). This hierarchical model provides a way to estimate covariate effects while controlling for variability in the form of state-specific random effects. Time (years) since 1999 was used to measure annual change and an interaction between time and each risk factor tested for whether the risk factor modified the rate of change. All models used two sided tests at $\alpha=0.05$ to determine significance.

Results

From 1999-2016, there were 700,300 incident cases of pancreatic cancer in the United States. Concurrently, 351,630 opioid overdose deaths occurred during this same period.(16)

There were marked differences in the distribution of pancreatic cancer incidence and opioid death rates among the states. However, both incidence and death rates

increased from 1999 to 2016 in most states (Fig. 1 and 2, Supplemental Fig. 1). The PC incidence rate in the average state increased at an unadjusted rate of 0.137 percentage points per year from 1999 to 2016. (Table 1)

Among contributing factors evaluated at a state level, the opioid death rate increased significantly over the study period (Fig. 2B, Beta β=.546, P-value < 0.0001). We also observed an increase in obesity prevalence (Supplement Fig. 3A, Beta β=.605, P-value < 0.0001). We observed no increase or decrease in alcohol prevalence (Supplement Fig. 3B) (β=-0.0003, P-value 0.994). Cigarette use prevalence decreased significantly over time (Supplement Fig. 3C) (β=-0.306, P-value < 0.0001). Values for each risk factor by state with confidence intervals are provided in the supplementary tables 1-5. Data plotted for each of these variables at a national level is shown in supplementary figure 2.

We employed a mixed-effects regression model to provide a multivariate analysis of the longitudinal associations between the potential risk factors (opioid use, obesity, alcohol and smoking) and the change in incidence rates of pancreatic cancer. Results of the mixed effect model are presented in Table 2. The risk factor adjusted PC rate increased over time by an average of 0.108 percentage points per year. The cross-sectional rate of PC was significantly related to the 4-year lagged opioid death rate (β=0.185, p<0.0001), while this effect got smaller over time (β=-.019, p<0.0001). Levels of alcohol use were also associated with higher rates of PC (β=0.025, p=0.007). Obesity rate was not associated with rate of PC (β=0.023, p=0.493), nor degree of change of PC rates (β=0.002, p=0.404). However, there was a significant interaction

between opioid death rate and obesity prevalence (β=0.017, p=0.0002). Indicating rates of PC were higher in states with elevated levels of both obesity and opioid usage 4 years prior.

Figure 2C illustrates the interaction of the opioid death rate with PC rates over time in states with varying level of opioid death rate at the beginning of the study. Although the overall trend in PC rates is increasing across the states, the rate of the increase is larger for those states starting at lower opioid levels and lower for those that started with higher levels of opioid usage (Fig. 2C).

Discussion

Our results indicate opioid usage, estimated by opioid death rates may partially explain the uptrend of PC rates in the U.S. at the national as well as the state levels over time (Fig. 2 and Supplemental Fig. 1). These findings suggest that opioids use may be a novel risk factor for PC, a finding that needs further studies. Once confirmed, implications of the opioids on PC development are clinically significant given the widespread opioid usage in the country. Furthermore, opioids are widely used for pain management in pancreatitis, an established precursor to PC (17, 18) as well as in the cancer itself (19).

We confirmed the increase in the PC rates over time with the majority of the states showing an uptrend in the disease rates over the period of our study (16 years). Opioid

use remained a significant predictor to the PC rates although its overall effect became weaker over time, suggesting a ceiling effect. This is supported by our observation that the most increase in PC rates over time occurred in the states that initially had the lowest opioid death rates and showed a significant increase in this opioid index by time (Fig. 2).

The association of obesity prevalence and the opioid index in the national databases may be explained by the observation that opioid usage is associated with a poorer lifestyle in general (20). Such populations could be exposed to other conditions and comorbidities associated with a poor lifestyle that did not enter our model. As suggested by the opposite trend in cigarette use prevalence and PC rates over time in U.S, cigarette use was not associated with PC rate in our analysis.

Opioids and its derivatives are widely used for the management of pain in PC. A large retrospective study found a negative correlation between opioid usage and survival time in patients with unresectable tumors (3). Population based studies from an area in Iran with high opium consumption rate proposed opium to increase risk of PC (7, 8). Furthermore a recent post hoc analysis of two randomized controlled trials of patients with advanced cancers (including pancreatic cancer), revealed that that those treated frequently with an opioid antagonist had significantly improved overall survival compared to placebo (21).

Mechanistically, opioids as a class of drugs, have been previously studied to have possible carcinogenicity in cancers other than PC (22). Multiple studies have shown that opioids have a potential to promote cancer progression and metastases in multiple different types of cancers including breast, prostate, lung, esophageal and hepatocellular cancer by various mechanisms including activation of the mTOR pathway, promoting angiogenesis and even promoting epithelial to mesenchymal transition (23). In addition opioids have been shown to alter the gut microbiome (24) which in turn has been shown to modulate pancreatic carcinogenesis (25). With these mounting data, the impact of opioids on pancreatic cancer formation and/or progression certainly needs to be further studied.

Using state-based datasets over 16 years, our data suggest opioid usage as a potential risk factor for pancreatic cancer. We used the publicly available data from the CDCs robust reporting system. However, we acknowledge limitations of our analysis. Opioid death rate was used a surrogate for overall opioid use. While prescription opioid use increased in the mid to late 1990s, nonmedical opioid use also increased significantly in the early 2000s and constitutes a large proportion of overall opioid use (26). Hence, we believe, unlike the opioid prescription rates that miss out the illicit and illegal usage, the opioid death rate index accounts for the pattern of both prescription as well as illicit opioid use across the states. The tested variables showed high variabilities among the fifty states with some missing values per the state-year. We used a mixed effects model to analyze repeated-measures data over time which has several advantages including accounting for the initial levels of cancer rate as sources of random variability and

allows for variation in number of observations across the groups (e.g., the states), allowing all possible data to be used. BRFSS data on obesity (based on height and weight) are self-reported and as such could be subject to misreporting. However, this data could be utilized for estimating trends over time reliably since the same source of self-reported data was consistently used for the trend calculations during the study period here.

In summary, our analysis of state-based databases suggests that opioid usage pattern may explain the trend of increased pancreatic cancer over time. Opioids as a novel risk factor pancreatic cancer needs to be confirmed in population-based studies.

Disclosure: None of the authors have any potential conflicts (financial, professional or personal) related to the manuscript to disclose.

References:

1. Khalaf N, El-Serag HB, Abrams HR, Thrift AP. Burden of Pancreatic Cancer-From Epidemiology to Practice. Clin Gastroenterol Hepatol. 2020.
2. Kleeff J, Korc M, Apte M, La Vecchia C, Johnson CD, Biankin AV, et al. Pancreatic cancer. Nat Rev Dis Primers. 2016;2:16022.
3. Oh TK, Do SH, Yoon YS, Song IA. Association Between Opioid Use and Survival Time in Patients With Unresectable Pancreatic Cancer: 10 Years of Clinical Experience. Pancreas. 2018;47(7):837-42.
4. Hidalgo M. Pancreatic cancer. N Engl J Med. 2010;362(17):1605-17.
5. Korc M, Jeon CY, Edderkaoui M, Pandol SJ, Petrov MS, Consortium for the Study of Chronic Pancreatitis D, et al. Tobacco and alcohol as risk factors for pancreatic cancer. Best Pract Res Clin Gastroenterol. 2017;31(5):529-36.
6. Ryan DP, Hong TS, Bardeesy N. Pancreatic adenocarcinoma. N Engl J Med. 2014;371(11):1039-49.
7. Moossavi S, Mohamadnejad M, Pourshams A, Poustchi H, Islami F, Sharafkhah M, et al. Opium Use and Risk of Pancreatic Cancer: A Prospective Cohort Study. Cancer Epidemiol Biomarkers Prev. 2018;27(3):268-73.
8. Shakeri R, Kamangar F, Mohamadnejad M, Tabrizi R, Zamani F, Mohamadkhani A, et al. Opium use, cigarette smoking, and alcohol consumption in relation to pancreatic cancer. Medicine (Baltimore). 2016;95(28):e3922.
9. Scholl L, Seth P, Kariisa M, Wilson N, Baldwin G. Drug and Opioid-Involved Overdose Deaths - United States, 2013-2017. MMWR Morb Mortal Wkly Rep. 2018;67(5152):1419-27.
10. Vowles KE, McEntee ML, Julnes PS, Frohe T, Ney JP, van der Goes DN. Rates of opioid misuse, abuse, and addiction in chronic pain: a systematic review and data synthesis. Pain. 2015;156(4):569-76.
11. U.S. Cancer Statistics Working Group. U.S. Cancer Statistics Data Visualizations Tool [Internet]. 2019, June. Available from: https://gis.cdc.gov/Cancer/USCS/DataViz.html.
12. Multiple Cause of Death 1999-2018 on CDC WONDER Online Database [Internet]. 2020 Available from: https://www.kff.org/other/state-indicator/opioid-overdose-death-rates/?activeTab=map&currentTimeframe=0&selectedDistributions=opioid-overdose-death-rate-age-adjusted&sortModel=%7B%22colId%22:%22Location%22,%22sort%22:%22asc%22%7D.
13. BRFSS Prevalence & Trends Data [Internet]. Available from: https://www.cdc.gov/brfss/brfssprevalence/index.html.
14. Chari ST, Kelly K, Hollingsworth MA, Thayer SP, Ahlquist DA, Andersen DK, et al. Early detection of sporadic pancreatic cancer: summative review. Pancreas. 2015;44(5):693-712.
15. Laird NM, Ware JH. Random-effects models for longitudinal data. Biometrics. 1982;38(4):963-74.
16. Overdose Deaths Involving Opioids, Cocaine, and Psychostimulants — United States [Internet]. 2015–2016. Available from: DOI: http://dx.doi.org/10.15585/mmwr.mm6712a1.

17. Nusrat S, Yadav D, Bielefeldt K. Pain and opioid use in chronic pancreatitis. Pancreas. 2012;41(2):264-70.
18. Wu BU, Butler RK, Chen W. Factors Associated With Opioid Use in Patients Hospitalized for Acute Pancreatitis. JAMA Netw Open. 2019;2(4):e191827.
19. Lahoud MJ, Kourie HR, Antoun J, El Osta L, Ghosn M. Road map for pain management in pancreatic cancer: A review. World J Gastrointest Oncol. 2016;8(8):599-606.
20. Abuse NIoD. Addressing the Opioid Crisis Means Confronting Socioeconomic Disparities. October 25, 2017
21. Janku F, Johnson LK, Karp DD, Atkins JT, Singleton PA, Moss J. Treatment with methylnaltrexone is associated with increased survival in patients with advanced cancer. Ann Oncol. 2016;27(11):2032-8.
22. Li JH, Lin LF. Genetic toxicology of abused drugs: a brief review. Mutagenesis. 1998;13(6):557-65.
23. Singleton PA, Moss J, Karp DD, Atkins JT, Janku F. The mu opioid receptor: A new target for cancer therapy? Cancer. 2015;121(16):2681-8.
24. Wang F, Roy S. Gut Homeostasis, Microbial Dysbiosis, and Opioids. Toxicol Pathol. 2017;45(1):150-6.
25. Vitiello GA, Cohen DJ, Miller G. Harnessing the Microbiome for Pancreatic Cancer Immunotherapy. Trends Cancer. 2019;5(11):670-6.
26. Phillips JK FM, Bonnie RJ. Trends in Opioid Use, Harms, and Treatment. Pain Management and the Opioid Epidemic: Balancing Societal and Individual Benefits and Risks of Prescription Opioid Use. National Academies Press (US): National Academies of Sciences, Engineering, and Medicine; Health and Medicine Division; Board on Health Sciences Policy; Committee on Pain Management and Regulatory Strategies to Address Prescription Opioid Abuse; July 13 2017

Figure Legends:

Figure 1: Incidence of pancreatic cancer (1999 and 2016), U.S Cancer Statistics Working group, and opioid death rate by state (1999 and 2016), CDC Wonder database (A) Pancreatic cancer incidence visualization by state in 1999, (B) Pancreatic cancer incidence visualization by state in 2016(C) Opioid death rate visualization by state in 1999, (D) Opioid death rate visualization by state in 2016

Figure 2: (A) Incidence rate of pancreatic cancer over time per 100,000 people, State incidence rate of pancreatic cancer through the years 1999 to 2016, (B) Incidence rate of opioid death overtime per 100,000 people, State incidence rate of opioid death through the years 1999 to 2016, (C) A state with an opioid death rate in the 25th percentile in 1999 had an annual increase in PC that was 34 percent faster than a state at the median opioid death rate and 3.3 times faster than a state with an opioid death rate in the 75th percentile.

Table 1: Individual Mixed effects regression model analysis results (annual change)

Table 2: Mixed effects regression model analysis results

Supplementary tables legends

Supplement Table 1: Pancreatic Cancer Incidence Rate by State, United States Cancer Statistics: Data Visualizations

Supplement Table 2: Opioid Death Rate by State, United States Cancer Statistics: Data Visualizations

Supplement Table 3: Obesity Prevalence by State (%), Behavioral Risk Factor Surveillance System (BRFSS)

Supplement Table 4: Alcohol Use Prevalence by State (%), Behavioral Risk Factor Surveillance System (BRFSS)

Supplement Table 5: Cigarette Use by State (%), Behavioral Risk Factor Surveillance System (BRFSS)

Supplementary figures legends

Supplement Figure 1: (A) Incidence rate of pancreatic cancer over time per 100,000 people, National incidence rate of pancreatic cancer through the years 1999 to 2016, (B) Incidence rate of opioid death overtime per 100,000 people, National incidence rate of opioid death rate through the years 1999 to 2016,

Supplement Figure 2: (A) National prevalence of obesity through the years 1999 to 2018, (B) National prevalence (%) of alcohol use through the years 1999 to 2018, (C) National prevalence (%) of tobacco use through the years 1999 to 2017

Supplement Figure 3: (A) State prevalence of obesity overtime, Prevalence of obesity by state through the years 1999 to 2018, (B) Prevalence of alcohol use by state through the years 1999 to 2018, (C) Prevalence of tobacco use by state through the years 1999 to 2017

Figure 1 A-D

Click here to access/download;Figure;fig 1 A-D.tif

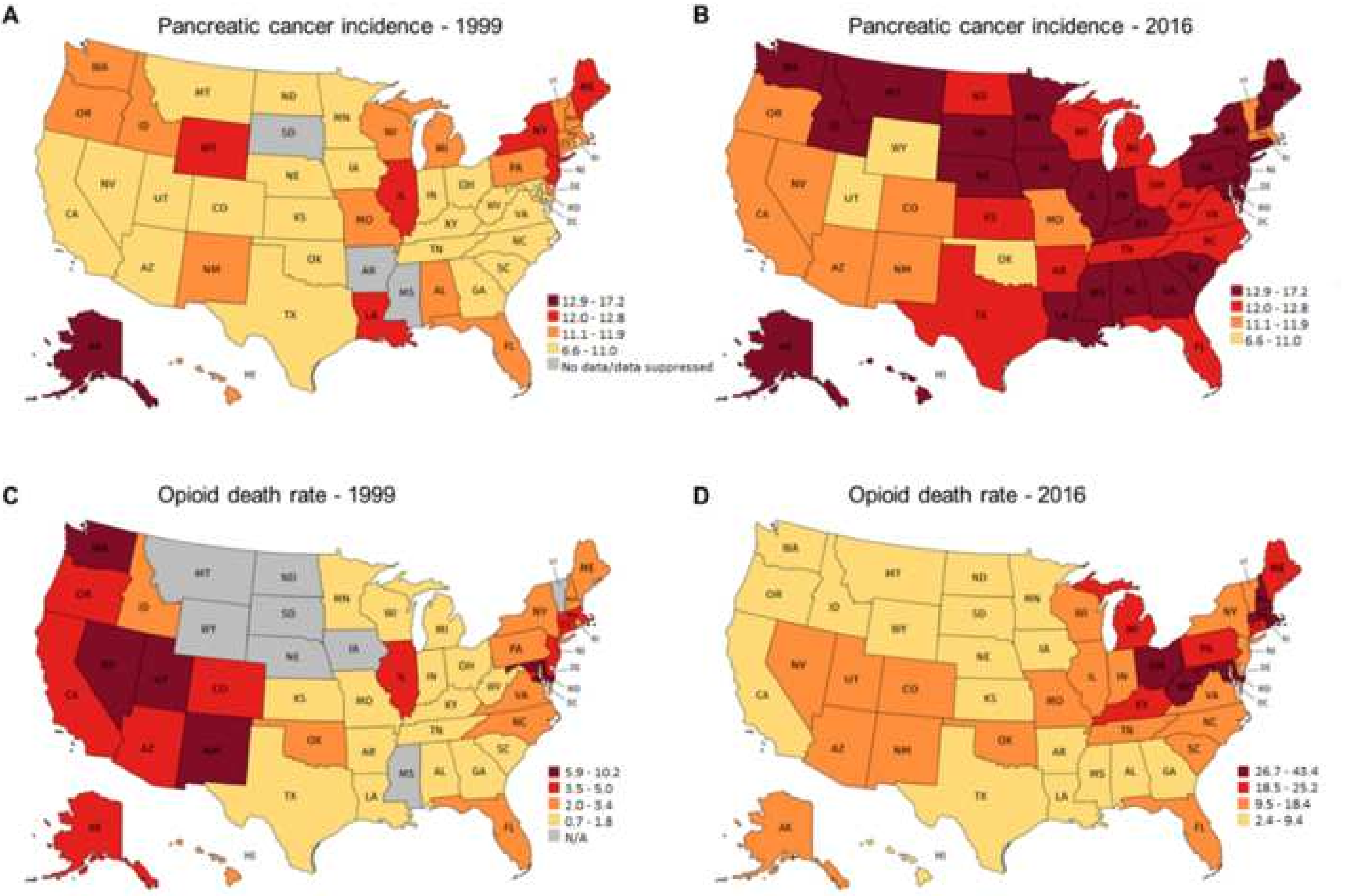

Figure 2 A-C

Click here to access/download;Figure;fig 2 A-C.tif

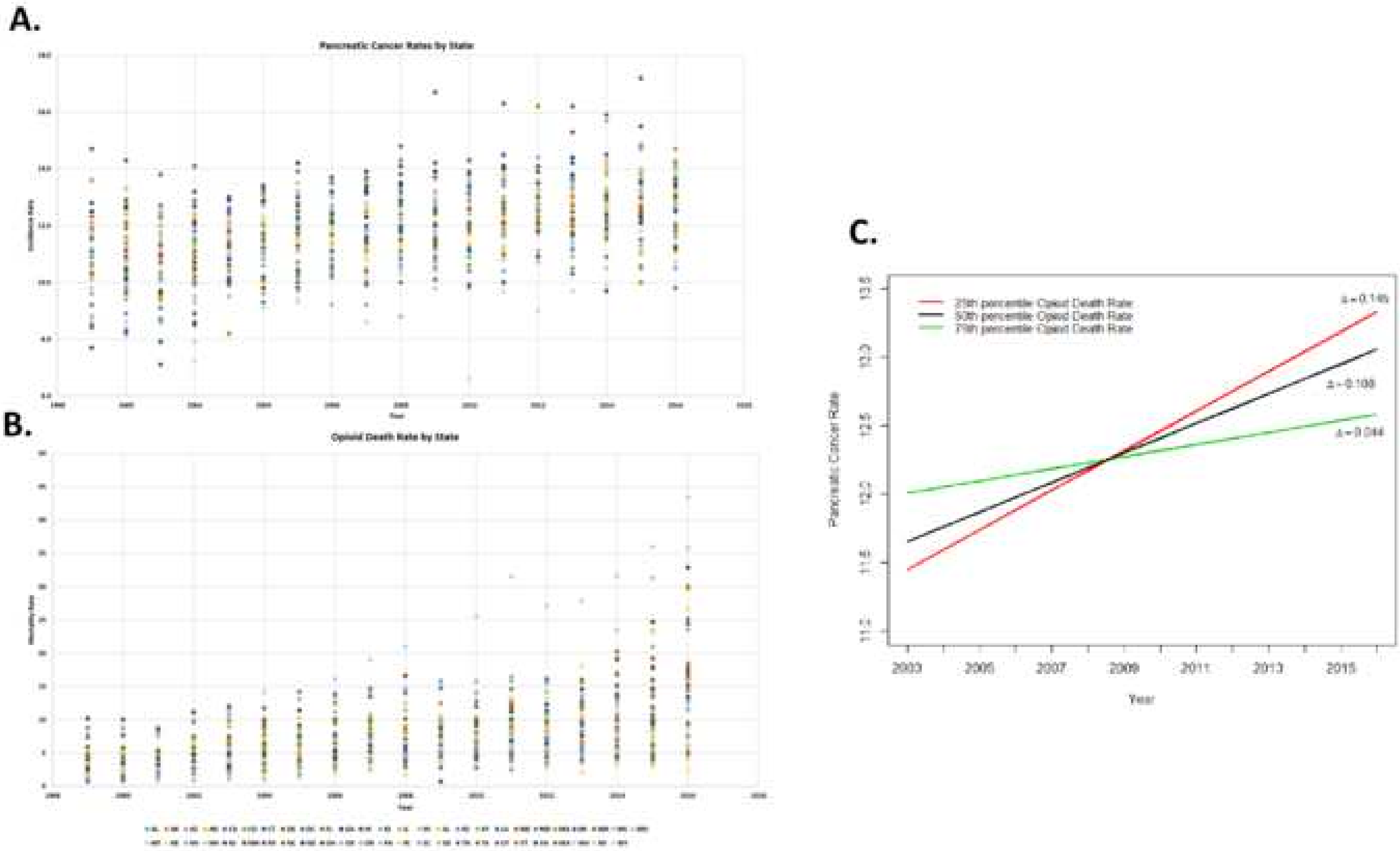

 Click here to access/download;Figure;Tables.docx

Table 1:

Individual Mixed Model (annual changes)	Estimate	Confidence Interval (95%)
Pancreatic cancer rate	0.137	0.119, 0.155
Opioid death rate	0.546	0.438, 0.653
Obesity prevalence	0.605	0.571, 0.639
Alcohol prevalence	-0.0003	-0.075, 0.074
Cigarette use prevalence	-0.306	-0.333, -0.279

Table 2:

Four-year lagged risk factors	Estimate	Standard Error	P-Value
Opioid death rate	0.185	0.047	< .0001
Obesity prevalence	0.023	0.033	0.493
Alcohol prevalence	0.025	0.009	0.007
Cigarette use prevalence	-0.014	0.018	0.429
Time	0.108	0.021	< .0001
**Interactions with risk factors with time**	**Estimate**	**Standard Error**	**P-Value**
Opioid death rate	-0.019	0.004	< .0001
Obesity prevalence	0.002	0.002	0.404
Interaction between opioid death rate and obesity prevalence	0.016	0.004	0.0002

Supplement Tables

Click here to access/download
Supporting Information
Supplement Tables.docx

Supplement Figure 1 A-B

Click here to access/download
Supporting Information
Supplement fig 1.tif

Supplement Figure 2 A-C

Click here to access/download
Supporting Information
supplement fig 2.tif

Supplement Figure 3 A-C

Click here to access/download
Supporting Information
supplement fig 3.txt
