# Supplementary material for: Opioid use as a potential risk factor for pancreatic cancer in the United States: An analysis of state and national level databases
Source: PLoS One. 2021 Jan 6;16(1):e0244285. doi: 10.1371/journal.pone.0244285 (PMC7787381; doi:10.1371/journal.pone.0244285)
Supplement: Attachment — Submitted filename: Response to Comments-Opiod-PDAC-Plos one.docx [file pone.0244285.s011.docx]

PONE-D-20-20071
Opioid use as a potential contributing factor to the rise of pancreatic cancer; an analysis of state and national level databases from the United States
PLOS ONE

Dear Dr. Bishehsari,

Thank you for submitting your manuscript to PLOS ONE. After careful consideration, we feel that it has merit but does not fully meet PLOS ONE’s publication criteria as it currently stands. Therefore, we invite you to submit a revised version of the manuscript that addresses the points raised during the review process.


Please submit your revised manuscript by Nov 06 2020 11:59PM. If you will need more time than this to complete your revisions, please reply to this message or contact the journal office at [plosone@plos.org](mailto:plosone@plos.org). When you're ready to submit your revision, log on to <https://www.editorialmanager.com/pone/> and select the 'Submissions Needing Revision' folder to locate your manuscript file.

Please include the following items when submitting your revised manuscript:

- A rebuttal letter that responds to each point raised by the academic editor and reviewer(s). You should upload this letter as a separate file labeled 'Response to Reviewers'.
- A marked-up copy of your manuscript that highlights changes made to the original version. You should upload this as a separate file labeled 'Revised Manuscript with Track Changes'.
- An unmarked version of your revised paper without tracked changes. You should upload this as a separate file labeled 'Manuscript'.

If you would like to make changes to your financial disclosure, please include your updated statement in your cover letter. Guidelines for resubmitting your figure files are available below the reviewer comments at the end of this letter.

If applicable, we recommend that you deposit your laboratory protocols in protocols.io to enhance the reproducibility of your results. Protocols.io assigns your protocol its own identifier (DOI) so that it can be cited independently in the future. For instructions see: [http://journals.plos.org/plosone/s/submission-guidelines#loc-laboratory-protocols](https://urldefense.com/v3/__http:/journals.plos.org/plosone/s/submission-guidelines*loc-laboratory-protocols__;Iw!!OlavHw!o2iScHKL_fjwZMocO-MLKTR_bJPV92mmQxjC8HsJbWDtvXQXzk0X_QyKkQj432ihLPNtfVU$)

Reviewers' comments:

Reviewer's Responses to Questions

Comments to the Author

1. Is the manuscript technically sound, and do the data support the conclusions?

The manuscript must describe a technically sound piece of scientific research with data that supports the conclusions. Experiments must have been conducted rigorously, with appropriate controls, replication, and sample sizes. The conclusions must be drawn appropriately based on the data presented.

Reviewer #1: Partly

Reviewer #2: Yes

________________________________________

2. Has the statistical analysis been performed appropriately and rigorously?

Reviewer #1: Yes

Reviewer #2: Yes

________________________________________

3. Have the authors made all data underlying the findings in their manuscript fully available?

The PLOS Data policy requires authors to make all data underlying the findings described in their manuscript fully available without restriction, with rare exception (please refer to the Data Availability Statement in the manuscript PDF file). The data should be provided as part of the manuscript or its supporting information, or deposited to a public repository. For example, in addition to summary statistics, the data points behind means, medians and variance measures should be available. If there are restrictions on publicly sharing data—e.g. participant privacy or use of data from a third party—those must be specified.

Reviewer #1: Yes

Reviewer #2: Yes

________________________________________

4. Is the manuscript presented in an intelligible fashion and written in standard English?

PLOS ONE does not copyedit accepted manuscripts, so the language in submitted articles must be clear, correct, and unambiguous. Any typographical or grammatical errors should be corrected at revision, so please note any specific errors here.

Reviewer #1: Yes

Reviewer #2: Yes

________________________________________

**Response:**

**We would like to thank the editor and the reviewers who found our study to have merits. Here we** **submit a revised version of the manuscript after addressing the points raised by the reviewers. We believe that the revision, as well as new analyses that we have done according to the reviewers’ suggestions, have improved the impact of our manuscript. Please find below our response to reviewers’ comments.**

5. Review Comments to the Author

Please use the space provided to explain your answers to the questions above. You may also include additional comments for the author, including concerns about dual publication, research ethics, or publication ethics. (Please upload your review as an attachment if it exceeds 20,000 characters)

Reviewer #1: The authors present results of a study using state and national level databases to assess the association between opioid use and pancreatic cancer. Using mixed effects models and state level data across 16 years of observation, authors find that opioid death rates are associated with incident pancreatic cancer rates 4 years later as well as change over time in the incident pancreatic cancer rates. They conclude that opioid use may account for some of the increase in pancreatic cancer. The manuscript will be strengthened if the authors consider the following points.

1. Authors are using estimated values from a variety of sources (and include 95% confidence intervals in the supplemental tables). Did the authors use the uncertainty in these values in any way in their analyses? At least some mention of this as a limitation would be good.

**We agree with the reviewer, that although the datasets that we used are reliable we cannot independently ascertain the extracted values. We added this as a limitation in the discussion (page 13, first paragraph).**

2. Were the assumptions of normality met for the outcomes evaluated with the mixed effects regression model? I am wondering about the opioid death rates in particular.

**Thank you for this insightful comment. We have now re-analyzed our data and reported our findings in the manuscript (page 9, 2^nd^ paragraph).** **The opioid death rate is not normally distributed; we re-ran the mixed effects model using the log transformation of opioid death rate. The output of the model has not changed, and still showed a strong statistically significant relationship between opioid death rate and pancreatic cancer incidence. All other significant variables were retained in this model from the previous one. We report these new results in a new supplement table 6 in our revision.**

**Additionally, all outcomes met normality assumptions for the mixed effects regression models. We are using pancreatic cancer incidence as our outcome, not opioid death rate.**

3. In the results, authors interpret the multivariate model results and in particular mention that obesity rate was not associated with the rate of pancreatic cancer. However, there is an interaction in the model (between obesity rate and opioid death rate), which makes the interpretation of the main effect not as straight forward. Authors should clarify their interpretation. Also, it is not clear from the table whether this interaction was an interaction with time. The coefficient in the text differs slightly than that in the table, so that should be corrected. The sentence in the text reporting that interaction and the sentence following it likely should be combined (as the last sentence is an incomplete sentence).

**We agree with the reviewer that our initial format of reporting the interaction results could have been confusing. We have made changes to the table to make the distinction that the interaction between obesity and opioid death rate was separate from the interaction with time. We have also mentioned that this interaction may be due to opioid use and obesity being associated with other comorbidities due to poor lifestyle (page 11, paragraph 2. We have also clarified our explanations of the statistical models in the method section.**

4. The authors used lagged risk factors to predict the rates of pancreatic cancer. Did authors consider assessing how change in opioid death rates were associated with change in pancreatic cancer rates?

**We would like to thank the reviewer for this comment. We clarified this issue in the revised manuscript both in the result section (page 9,last paragraph) and the discussion (page 11, first paragraph). Specifically, Figure 2C addresses this issue where we show how states in the 25^th^ percentile of opioid death rates had the largest increases in the pancreatic cancer rate while states in the 75^th^ percentile of opioid death rates had slower increases in the pancreatic cancer rates.**

5. Do the authors have any comments about the limitations of ecologic analyses in the context of their question?

**We do agree with the reviewer that this is a limitation of our study, and expanded on this limitation in our discussion (page 13, last paragraph).**

Minor points:

1. In the abstract, the authors give the result of how the opioid death rate predicted incident pancreatic cancer. Authors might want to clarify here that they are predicting incident pancreatic cancer 4 years later. Also, in the next line, authors talk about the significant effect on the estimated annual change. The earlier sentence refers to the initial level of a state's opioid death rate, but they appear to use the time-varying 4-year lagged variable, so the effect on change over time is not restricted to just the initial level.

Suggested Change:

The initial level of a state’s opioid death rate A state’s opioid death rate at 4 years prior significantly predicted initial incidence of PC (β=0.1848, p<0.0001) and had a significant effect on the estimated annual change in the rate of PC (β=-.0193,p<0.0001).

**We would like to thank this reviewer for this comment. We have made the suggested changes to the abstract which we believe has made it clearer and more concise.**

2. Under Data collection: "This data was" should be "These data were" and authors refer to Tables 1-5, but I think they mean Supplemental Tables 1-5. ("this data" also appears in the Discussion section and should "these data").

**Thank you for this correction, this phrase has now been corrected in the manuscript.**

3. Figure 1: Panels C and D use the same colors, but they have different meanings. Since authors likely want readers to visually compare the two graphs to see the changes in opioid death rates, authors should consider having the same rate categories and colors (where applicable) and then have additional categories/colors to more easily see the differences.

**We appreciate this detailed feedback. We have improved the map figure. It now shows the same colors for each category to help keep each figure consistent for the viewer.**

Reviewer #2: the highlighted text should be edited and rewrite. Also, potential ecologic fallacy is one of the limitations of the current study that should be addressed in the discussion. figures in the text had not good resolution.

**We would like to thank this reviewer for the helpful comments on our manuscript. We have now addressed the first point in the discussion of the manuscript where we discussed potential ecologic fallacy of our study (page 13, last paragraph). We have also increased the quality of the map figures.**
